# Comparative transcriptomic profiles of *Paulownia catalpifolia* under different degrees of chilling stress during the seedling stage

**DOI:** 10.1186/s12864-024-10613-7

**Published:** 2024-07-24

**Authors:** Baojun Liu, Jiang Su, Chuanming Fu, Kanghua Xian, Jinxiang He, Ningzhen Huang

**Affiliations:** Guangxi Key Laboratory of Plant Conservation and Restoration Ecology in Karst Terrain, Guangxi Institute of Botany, Guangxi Zhuang Autonomous Region and Chinese Academy of Sciences, Guilin, 541006 China

**Keywords:** *Paulownia catalpifolia*, Chilling stress, Transcriptomic profile, Physiologic index, Differentially expressed genes

## Abstract

**Background:**

*Paulownia*, an ecologically and economically valuable plant species native to China, is notable for its excellent timber quality and strong adaptability. Among them, *Paulownia catalpifolia* displays the ability to survive in cold climate, a trait associated with northern China. Yet, the molecular information for its cold-tolerance has not been explored. This study was to investigate the changes in physiological indices and transcript levels of *P. catalpifolia* following cold exposure, which could provide evidence for revealing whether there were differences in the genetic basis of inducing physiological perturbations between moderate low temperature (MLT) and extreme low temperature (ELT).

**Results:**

The detection of physiological indices under diverse degrees of chilling stress showed similar patterns of alteration. Enhanced accumulation of osmoregulatory substances, such as soluble sugar and soluble protein, were more conducive under ELT compared to MLT in *P. catalpifolia*. Moreover, we observed leaf wilting symptoms distinctly after exposure to ELT for 48 h, while this effect was not obvious after MLT exposure for 48 h. Comparative transcriptomic analysis between MLT and ELT demonstrated 13,688 differentially expressed genes (DEGs), most of them appeared after 12 h and 48 h of treatment. GO and KEGG analyses elucidated prominent enrichment in aromatic-L-amino-acid decarboxylase activity term and carbohydrate metabolism pathways. Therefore, it was speculated that the DEGs involved in the above processes might be related to the difference in the contents of soluble protein and soluble sugar between MLT and ELT. Time series clustering analyses further highlighted several key genes engaged in the ‘Glycosyltransferases’, ‘Galactose metabolism’ and ‘Starch and sucrose metabolism’ pathways as well as the ‘tyrosine decarboxylase activity’ term. For instance, *cellulose synthase-like A* (*CLSA2*/*9*), *raffinose synthase* (*RafS2*), *β-amylase* (*BAM1*) and *tyrosine*/*DOPA decarboxylase* (*TYDC1*/*2*/*5*) genes, diverging in their expression trends between MLT and ELT, might significantly affect the soluble sugar and soluble protein abundance within *P. catalpifolia*.

**Conclusion:**

Between MLT and ELT treatments, partial overlaps in response pathways of *P. catalpifolia* were identified, while several genes regulating the accumulation of osmotic adjustment substances had disparate expression patterns. These findings could provide a novel physiological and molecular perspective for *P. catalpifolia* to adapt to complex low temperature habitats.

**Supplementary Information:**

The online version contains supplementary material available at 10.1186/s12864-024-10613-7.

## Background

Low temperature serves as a significant environmental challenge, which negatively impacting the growth of various plant species, thereby affecting their quality and geographic distribution [1]. Low temperature can lead to the degradation of lipids, proteins and DNA, subsequently causing cellular death. Typical symptoms of chilling damage include stunted growth and development, excessive production of reactive oxygen species (ROS), as well as disruptions in chloroplast integrity and photosynthetic electron transport. Cold stress can be classified into chilling stress (0–15℃) and freezing stress (< 0℃). Generally, plants from temperate climatic zones can enhance their cold tolerance by exposure to non-freezing low temperature, designated as cold acclimation [2]. Following cold hardening, plants can acquire complete tolerance, break dormancy and resume regular growth once the conditions are favorable. From a molecular perspective, cold acclimation involves the activation of cold-responsive genes that play vital roles in stabilizing cell membrane function, modulating lipid formation, and enhancing antioxidant enzyme activity to resist chilling injury. The main genes in this process include late embryogenesis abundant (LEA) proteins, heat shock proteins (HSP), and pathogenesis-related (PR) proteins, which establish a relationship between biotic and abiotic stress [3, 4].

The interaction between cold stress signals and other signaling pathways plays a vital role in maintaining the balance between stress responses and plant growth. These pathways incorporate phytohormones (like gibberellin, auxin and cytokinin), circadian rhythm, photosynthesis and floral transition. Cold tolerance, a complex quantitative trait, is regulated by multiple genes within the low-temperature signal transduction network [1, 5]. These genes can be divided into two distinct functional categories: (1) encoding proteins that safeguard cells from low temperature, including osmoregulation substances (such as soluble sugar), ROS scavenging enzymes (including peroxidase), and other protective macromolecules (like LEAs); (2) encoding regulatory proteins such as transcription factors, protein kinases and phosphatases [3, 6].

The functional genes, which particularly involved in sugar metabolism and antioxidant enzyme activity, are crucial for cold tolerance in plants. The key enzymes in sugar metabolism, such as sucrose synthase (SS), sucrose phosphorylase (SP), galactinol synthase (GOLS), sucrose phosphate synthase (SPS), raffinose synthase (Rafs), stachyose synthase (STS) and trehalose 6-phosphate phosphatase (TPP), had been characterized in *Camellia oleifera* [7]. Moreover, plants deployed enzymatic and non-enzymatic antioxidants to effectively scavenge oxidative stress induced by low temperatures. Enzymatic antioxidants such as ascorbate peroxidase (APX), superoxide dismutase (SOD) and catalase (CAT) exhibited regulatory activity in response to chilling stress [8]. The DREB1/CBF transcription factor (TF), which belonged to the AP2/ERF family, was also essential for cold response. It was first discovered in Arabidopsis as the regulatory gene, and could be rapidly induced upon cold exposure [9, 10]. To date, many TFs responsive to multiple abiotic stresses, such as drought, low temperature, heat shock, high salinity and abscisic acid (ABA), which contained the members from diverse families like AP2/ERF, MYC/MYB, HSF, bZIP, WRKY, bHLH, ZFP, NAC and AREB/ABF, had been identified in various plants [5, 11].

Several studies had focused on the different response pathways of transcription factors and functional genes between two degrees of low temperature. Two members of interest in these studies were the transcription factor gene *ICE1* and the functional gene *PAL*. ICE1, a confirmed member of bHLH TF family, might play a critical role in regulating *CBF* expression. Similar to Arabidopsis *AtICE1*, the *CdICE1* gene from *Chrysanthemum dichrum* could activate the expression of *CBFs* and their downstream cold-regulated genes when the temperature fell from 23℃ to 4℃. Interestingly, when transferred from 23℃ to 16℃, *CdICE1* appeared to enhance the chilling tolerance of transgenic Arabidopsis partially via the *miR398-CSD* pathway. These findings suggested that the *CdICE1*-mediated low temperature response pathways might be different, implicating *CBF* or *miR398*, depending on whether the cold acclimation was at 4℃ or 16℃ [12]. Furthermore, L-phenylalanine ammonia-lyase (PAL), a widely acknowledged environmental stress marker, exhibited distinctive responses under different degrees of chilling stress. Comparative analysis of *PAL* expression level in *Citrus clementina* fruits at 2℃ and 12℃ revealed a unique mRNA accumulation during 2℃ exposure, contrasted by a transient increase in *PAL* transcript levels at 12℃, which declined after 1 d [13]. These results indicated a stronger response under 2℃ compared to 12℃. Therefore, investigating whether there were differences in the expression trends of specific genes between different degrees of chilling stress might offer a novel perspective for understanding molecular mechanism of plant cold response.

*Paulownia*, as a member of the Paulowniaceae family, has a cultivation history spanning over 2000 years in Asia. The main habitat is located south of Beijing in mainland China, while its propagation extends to other Asian regions, including Myanmar and Vietnam. Different *Paulownia* species demonstrate wide adaptability to different temperature ranges, which is significantly related to the climatic conditions of their geographical habitats. The sub-zero or near-zero low temperature during the active growth stage, is a challenge for non-defoliated seedlings, and often leads to severe damage. However, these plants display an impressive recovery capacity, and can resume growth within a few weeks after leaf abscission. Among these *Paulownia* species, *P. catalpifolia*, *P. tomentosa* and *P. elongate* were found in the north of the Yangtze River, while *P. fortunei*, *P. australis*, *P. kawakamii* and *P. fargesii* were more common in the south of China [14]. Furthermore, the above *Paulownia* genus exhibited different cold tolerance. *P. tomentosa* could endure temperatures as low as -20℃, followed by *P. elongata* and *P. catalpifolia* with their ability to withstand − 15℃ to -18℃, whereas *P. fortunei*, *P. australis*, *P. kawakamii* and *P. fargesii* could survive in -5℃ to -10℃ [15]. Nevertheless, *P. tomentosa* was easy to branch excessively when growing in open spaces [16], and the growth rate of which was slow. In contrast, *P. catalpifolia*, known for its straight trunk, high density and attractive texture [17], exhibited a slightly faster growth rate than *P. tomentosa*. Consequently, given a balance of growth rate, wood trait and cold tolerance, we chose *P. catalpifolia* as the object of this study.

In fact, the adult tree of *P. catalpifolia* thrived well in cold zones of northern China [15]. In an attempt to reveal the complexity of the gene regulatory network of *P. catalpifolia* in response to differential chilling degrees, two low temperatures of 15℃ (moderate low temperature, MLT) and 5℃ (extreme low temperature, ELT) were conducted. Transcriptome reprogramming of *P. catalpifolia* potted seedlings under MLT and ELT stresses was investigated by RNA-seq technology. Comprehensive analysis of physiological indices and transcript levels suggested that the majority of differentially expressed genes (DEGs), showing the enrichment in the ‘Glycosyltransferases’, ‘Galactose metabolism’ and ‘Starch and sucrose metabolism’ pathways, along with the ‘tyrosine decarboxylase activity’ term, demonstrated disparate expression patterns. This research could further understand the vital pathways and DEGs, which might lead to physiological dissimilarities between differing chilling degrees in the leaves of *P. catalpifolia*, as well as help to provide potential candidate genes for the creation of cold-tolerant germplasm of *Paulownia*.

## Results

### Phenotypic and physiological responses under chilling stress

**Fig. 1:**
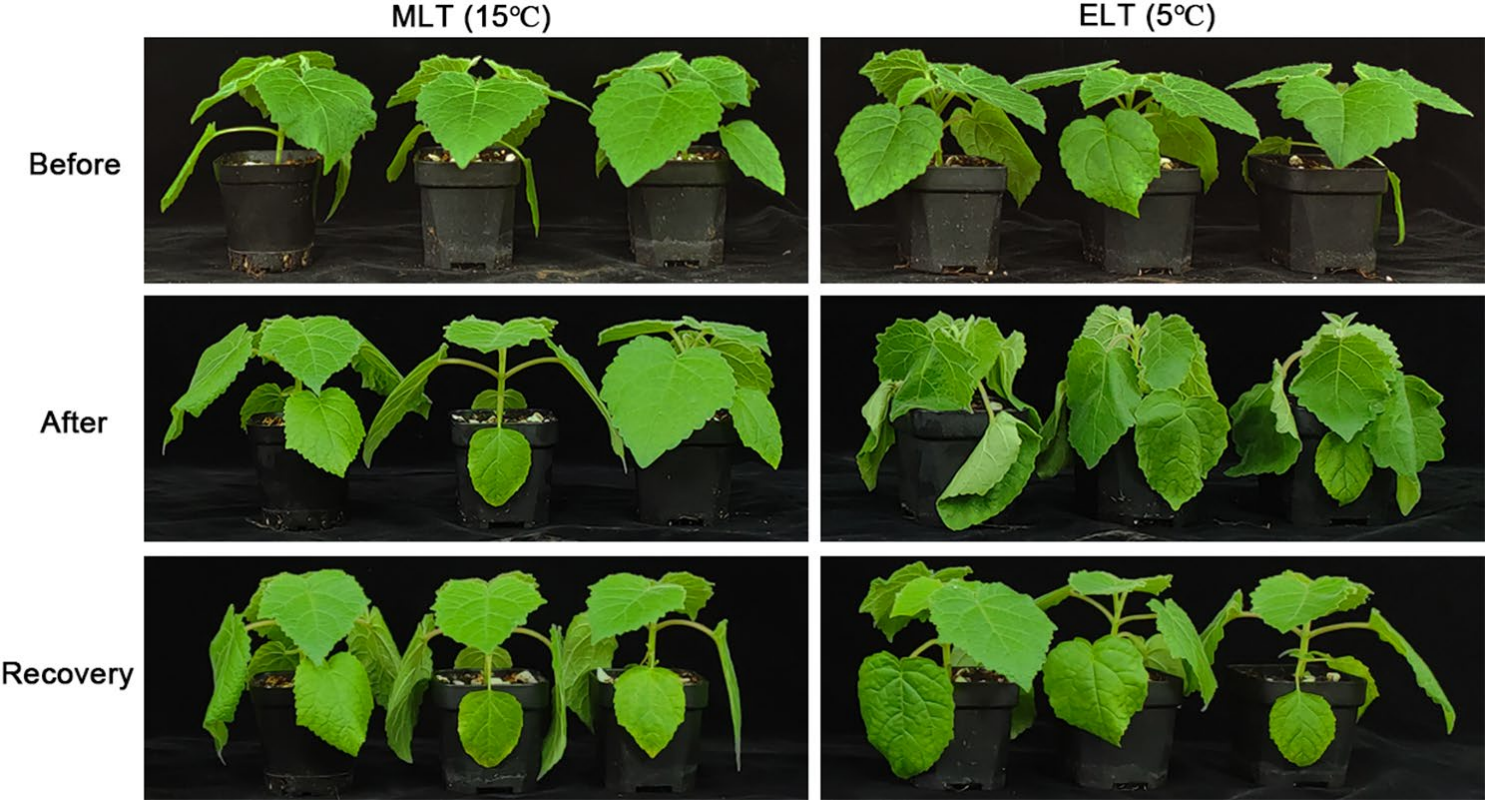
The morphological performances of the *P. catalpifolia* potted seedlings under MLT (15℃) and ELT (5℃) were shown on the left and right sides, images of seedlings were collected before cold treatment (**Before**), 48 h after cold treatment (**After**) and 48 h after recovery growth (**Recovery**), respectively

Two low temperature thresholds were set as 15℃ (moderate low temperature, MLT) and 5℃ (extreme low temperature, ELT). *P. catalpifolia* potted seedlings displayed distinctive morphological traits between MLT and ELT treatments. There was no significant morphological alteration in MLT. However, the phenotype of leaf wilting became apparent after 48 h of ELT. Following 48 h recovery period (R48 h), the leaves resumed their regular morphology. In general, compared with MLT, the potted seedlings exhibited more obvious morphological alterations similar to water deficit phenotype (Fig. 1), evolving to a semi-dormant state after 48 h of ELT.

**Fig. 2:**
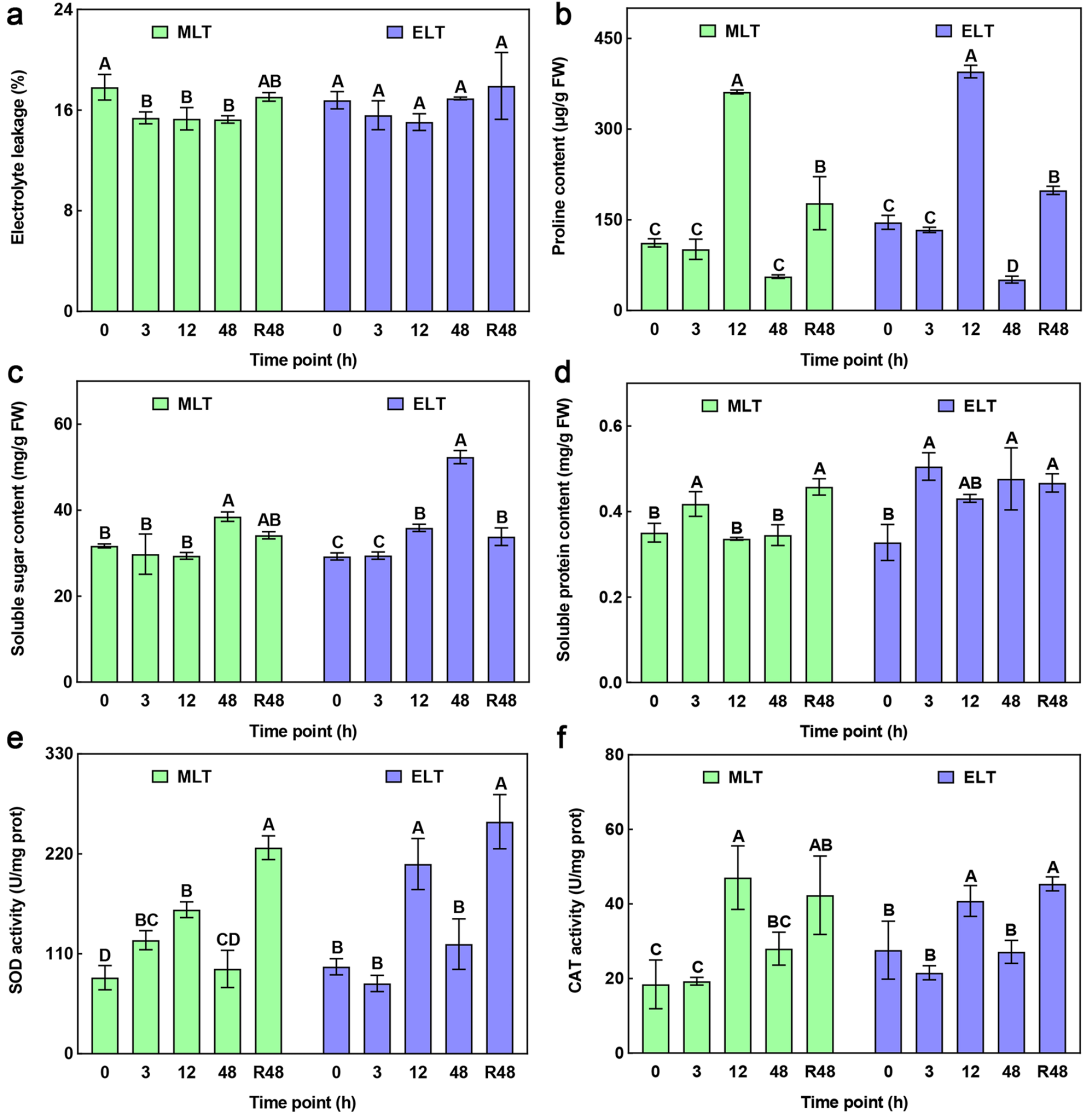
Effects of chilling stress on physiological characteristics of *P. catalpifolia* leaves were evaluated during MLT (green) and ELT (purple) period. The x-axis with 0, 3, 12, 48 and R48 indicated the time point (unit is h) after chilling treatment for 0 h, 3 h, 12 h, 48 h, and after recovery for 48 h, respectively. The y-axis represented corresponding values of physiological indices. **a** Relative electrolyte leakage (REC); **b** Proline content (PRO); **c** Soluble sugar content (SS); **d** Soluble protein content (SP); **e** Superoxide dismutase (SOD) activity; **f** Catalase (CAT) activity. Error values indicated the mean ± standard deviation calculated from three biological replicates, different capital letters **A**-**E** denoted significant differences (Duncan, *P* < 0.01). **FW**: Fresh weight; **prot**: protein

The physiological perturbation of young leaves from *P. catalpifolia* was detected under MLT and ELT. During MLT period, a declining trend in relative electrolyte leakage (REC; Fig. 2a) was observed, after 48 h recovery phase, REC returned to the same level as 0 h. The contents of proline (PRO; Fig. 2b) and soluble protein (SP; Fig. 2d), as well as the activities of superoxide dismutase (SOD; Fig. 2e) and catalase (CAT; Fig. 2f) showed an initial increase followed by a decrease. Nevertheless, after 48 h recovery phase, the above four physiological parameters obviously surpassed their levels at 0 h. The soluble sugar (SS; Fig. 2c) content only saw a substantial increase after 48 h of MLT. For the duration of ELT, there was no remarkable alteration in REC, while most other physiological indices followed similar patterns to those during MLT period, with a few variations at specific time intervals. For instance, compared with MLT, the PRO content displayed a significant decrease, while the SP content showed a marked increase after 48 h of ELT. Furthermore, after 12 h of ELT treatment and 48 h of recovery growth, SS content displayed a significant upward trend. In conclusion, the physiological parameters of P. *catalpifolia* potted seedlings experienced substantial perturbations under two chilling stresses.

In contrast to the decreasing pattern of REC under MLT, REC under ELT did not have a similar downward trend, but remained stable, indicating more serious cellular membrane damage under ELT. Additionally, fold changes in the other physiological parameters diverged between MLT and ELT treatments when compared to the initial point (0 h). In particular, the fold changes in SS contents (MLT: 0.9393-, 0.9267- and 1.2143-fold; ELT: 1.0073-, 1.2278- and 1.7911-fold), along with SP contents (MLT: 1.1918-, 0.9598- and 0.9840-fold; ELT: 1.5417-, 1.3143- and 1.4541-fold) at each time interval (3 h, 12 h and 48 h) in ELT were more prominent than those in MLT, respectively. These findings suggested that extreme chilling stress might be more conducive to the accumulation of osmoregulation substances in *P. catalpifolia*, such as soluble sugars and soluble proteins compared to moderate chilling stress.

### Sequencing statistics

To generate a reference transcriptome for *P. catalpifolia*, a total of 36 cDNA libraries of leaf samples collected at four intervals (0 h, 3 h, 12 h and 48 h) under the settings of NT (25℃), MLT (15℃) and ELT (5℃), each with three biological replicates, were constructed. Our existing dataset derived from previously unpublished work contributed twelve samples (NT), while the remaining 24 samples (MLT and ELT) were obtained from this study. Transcriptome sequencing was performed using the Illumina HiSeqTM 2000 sequencing platform. After filtration, a total of 14.79 billion clean reads was yielded, encompassing approximately 144.06 Gb of clean data, with an average data output of 6.00 Gb per sample. The values of Q30 and GC for each sample exceeded 91.03% and 46.9%, respectively. Due to the absence of a reference genome for *P. catalpifolia*, the Trinity software was employed for the *de novo* assembly of clean reads, resulting in 79,874,648 bases. This process generated a sum of 106,759 Trinity genes and 222,910 transcripts, with the median, average, and N50 length of contigs were 340 bp, 748.18 bp and 1,581 bp, respectively (Additional file 1: Table S1). The BLAST search conducted on the final set of unigenes against protein databases, revealed that 34,940 (32.73%), 34,697 (32.50%), 25,257 (23.66%), 18,040 (16.90%) and 17,975 (16.84%) of them were found to be matched with GO, UniProt, Nr, Pfam and KEGG databases, respectively (Additional file 1: Table S2). Moreover, a total of 39,949 unigenes (37.42%), returned at least one hit in blastX, whereas the remaining 66,810 unigenes were not matched (E-value < e-5), indicating the presence of many unknown genes or *Paulownia*-specific genes.

In order to reveal the effect on transcript components with the strengthening of chilling stress, we selected 24 samples under moderate low temperature (MLT0/3/12/48) and extreme low temperature (ELT0/3/12/48) for comparative analysis. For calculating the expression level of assembled transcripts, a total of 984.02 million clean reads were then mapped to the reference transcriptome, and the percent of mapped reads ranged from 64.79 to 71.86% (Additional file 1: Table S3). In the correlation heatmap, a deeper red shade indicated better repeatability within each group. The Pearson correlation coefficients (R^2^) varied from 0.984 to 0.997 (MLT0), 0.983 to 0.996 (MLT3), 0.99 to 0.996 (MLT12), 0.99 to 0.996 (MLT48), 0.974 to 0.99 (ELT0), 0.997 (ELT3), 0.952 to 0.985 (ELT12) and 0.993 (ELT48), respectively, reflecting a high level of consistency (Additional file 1: Fig. S2A). Principal component analysis (PCA) was performed to provide an overview of gene expression in the 24 cDNA libraries. The first two principal components contributed to approximately 95.67% of the total variability within the dataset. There were significant divergences in gene expression between two sets of chilling stress-treated samples and their respective controls. Yet, there was no evident separation between MLT3 and ELT12, as well as MLT12 and MLT48 samples, suggesting a similar expression pattern among them (Additional file 1: Fig. S2B).

### Differentially expressed genes during chilling stress period

**Fig. 3:**
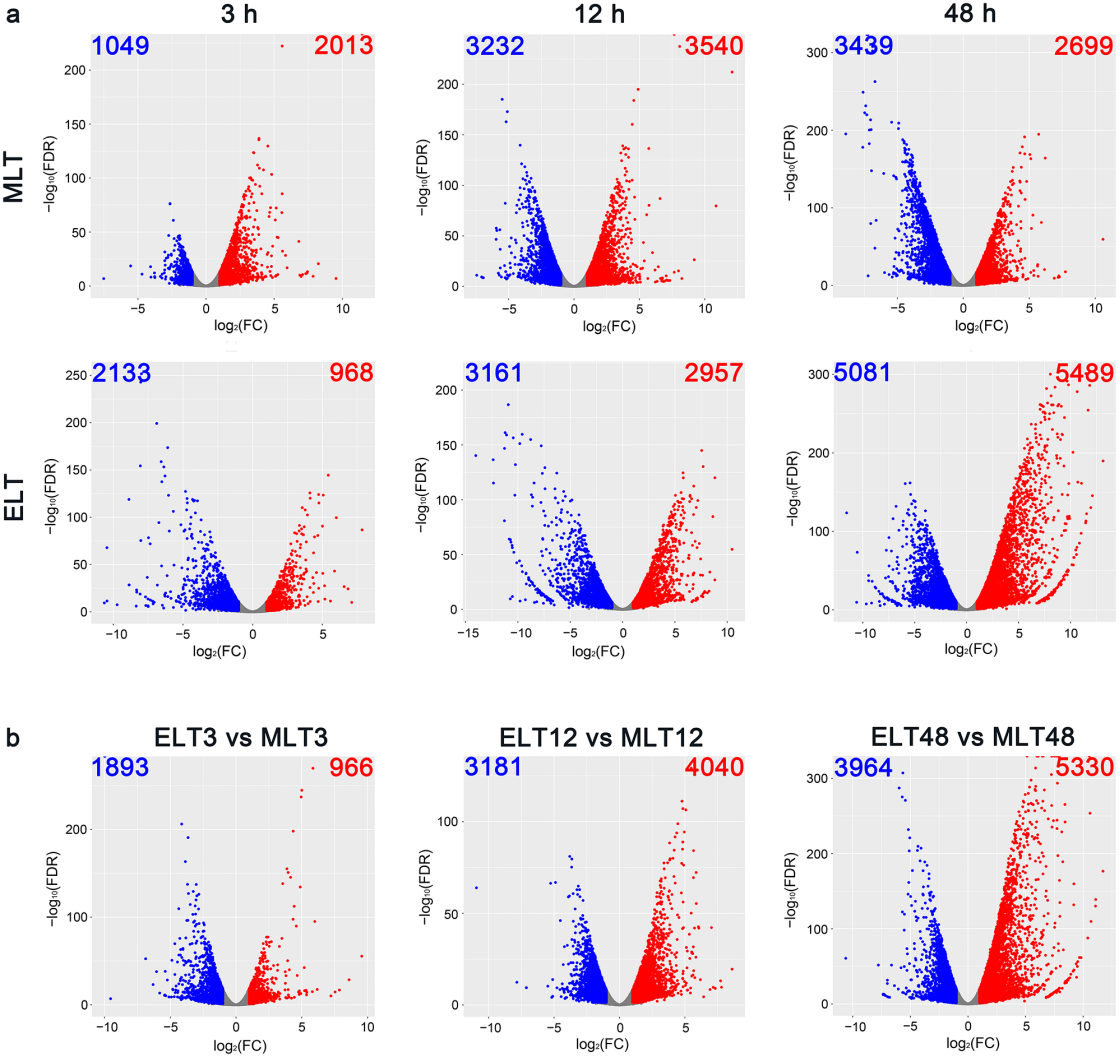
Volcano plots illustrated the differentially expressed genes (DEGs) in response to different degrees of chilling stress. The nine comparative pairs were designated by unique rectangles. **a** From left to right, the first row compared MLT3, MLT12 and MLT48 against MLT0, respectively. The second row performed the same for ELT3, ELT12 and ELT48 against ELT0, respectively; **b** From left to right, the third row represented ELT3 vs. MLT3, ELT12 vs. MLT12 and ELT48 vs. MLT48. In these diagrams, a single point corresponded to a distinct gene. The x-axis denoted the logarithm of the difference in gene expression (log_2_ FC) within a comparative pair, whereas the y-axis displayed the negative logarithm of the statistically significant gene expression difference (-log_10_ FDR). The number of down-regulated (blue) and up-regulated (red) DEGs were noted in the upper left and right corners of each rectangle. Genes with no significant differences in expression were represented by the gray points, without any count listed. FC: fold change; FDR: false discovery rate, equated with the adjusted *P*-value here

In our exploration of the changes in transcriptional abundance of *P. catalpifolia* leaves during MLT and ELT periods, differentially expressed genes (DEGs) in short-, medium- and long-term stress responses were evaluated. The identification of DEGs was performed by applying |log_2_ FC ≥ 1| and FDR ≤ 0.05, to the pairwise comparisons between the control (0 h) samples and chilling stress-treated samples (Additional file 2). The employed volcano plot could elucidate the dynamics of up-regulated and down-regulated DEGs. Six comparative pairs were established by considering MLT0 and ELT0 as control points across distinct time intervals. Of these, 3,062 (2,013 up-/1,049 down-regulated), 6,772 (3,540 up-/3,232 down-regulated) and 6,138 (2,699 up-/3,439 down-regulated) DEGs were distinguished in MLT3, MLT12 and MLT48 against MLT0, respectively. Meanwhile, we also identified 3101 (968 up-/2133 down-regulated), 6118 (2957 up-/3161 down-regulated) and 10,570 (5489 up-/5081 down-regulated) DEGs in ELT3, ELT12 and ELT48 against ELT0, respectively (Fig. 3a). During the initial 3 h of chilling stress (short-term), both MLT and ELT demonstrated a minimum count of DEGs, while the number of DEGs reached to peak for MLT stress at 12 h (medium-term) and for ELT stress at 48 h (long-term). Except for 12 h, the DEG counts of ELT surpassed those of MLT at other time points after cold exposure. These findings indicated that the transcriptional components of *P. catalpifolia* experienced more significant alterations with the prolongation of chilling stress duration and the strengthening of chilling stress degree.

GO (Gene Ontology) enrichment analysis was conducted to explore the biological roles of DEGs, with an emphasis on the top 20 significantly enriched GO terms (Additional file 3). Between different time points of MLT, there were predominant overlaps of down-regulated DEGs in the enriched GO terms, principally in ‘protein-chromophore linkage (GO:0018298)’ and ‘monoterpenoid biosynthetic process (GO:0016099)’ for the biological process (BP) category, as well as ‘chloroplast thylakoid membrane (GO:0009535)’ and ‘photosystem II (GO:0009523)’ within the cellular component (CC) category, along with ‘chlorophyll binding (GO:0016168)’ and ‘pigment binding (GO:0031409)’ from the molecular function (MF) category. While during ELT period, the main intersection occurred in enriched GO terms such as ‘protein-chromophore linkage (GO: 0018298)’ and ‘photosynthesis, light harvesting in photosystem I (GO: 0009768)’ (BP), ‘anchored component of plasma membrane (GO: 0046658)’ and ‘plant-type cell wall (GO: 0009505)’ (CC), as well as ‘chlorophyll binding (GO: 0016168)’ (MF). The considerable down-regulated gene overlaps were observed between MLT and ELT stresses, pointing towards a significant negative effect on chloroplast/plasma membrane and chlorophyll binding capacity, leading to inhibition of photosynthesis in *P. catalpifolia* potted seedlings under chilling stress. For up-regulated DEGs, during MLT period, primary intersections were ‘response to stress (GO:0006950)’, ‘thiamine biosynthetic process (GO:0009228)’ and ‘cellular amino acid metabolic process (GO:0006520)’ (BP), along with ‘aromatic-L-amino-acid decarboxylase activity (GO:0004058)’ and ‘tyrosine decarboxylase activity (GO:0004837)’ (MF) terms. Correspondingly, major intersections were identified with ‘thiamine biosynthetic process (GO:0009228)’, ‘response to chitin (GO:0010200)’, ‘raffinose family oligosaccharide biosynthetic process (GO:0010325)’, ‘galactose metabolic process (GO:0006012)’ and ‘carbohydrate storage (GO:0052576)’ (BP), in addition to ‘DNA-binding transcription factor activity (GO:0003700)’ and ‘inositol 3-alpha-galactosyltransferase activity (GO:0047216)’ (MF) under ELT treatment. There were differences in several GO terms between MLT and ELT among the up-regulated genes, with a remarkable enrichment of ‘aromatic-L-amino-acid decarboxylase activity’ (MF) in MLT whereas ‘carbohydrate storage’ (BP) in ELT. These findings suggested that the *P. catalpifolia* potted seedlings could effectively enhance the metabolic enzyme activities of aromatic-L-amino-acid or carbohydrate, as well as promote vitamin biosynthesis under either MLT or ELT treatment.

To identify the pathways activated by chilling stress, KEGG (Kyoto Encyclopedia of Genes and Genomes) enrichment analysis was conducted (Additional file 4), with a focus on the top 20 significantly enriched KEGG pathways. During the MLT phase, down-regulated DEGs mainly shared pathways like ‘Monoterpenoid biosynthesis (ko00902)’, ‘Photosynthesis-antenna proteins (ko00196)’, ‘Photosynthesis proteins (ko00194)’, ‘Cytochrome P450 (ko00199)’, ‘Methane metabolism (ko00680)’ and ‘Photosynthesis (ko00195)’. However, the down-regulated DEGs were primarily concentrated in the pathways including ‘Glycosyltransferases (ko01003)’, ‘Plant hormone signal transduction (ko04075)’, ‘Photosynthesis proteins (ko00194)’, ‘Photosynthesis (ko00195)’, ‘Photosynthesis-antenna proteins (ko00196)’ along with ‘Pentose and glucuronate interconversions (ko00040)’ during ELT period. Such findings elucidated most DEGs involved in carbohydrate/energy metabolism and signal transduction experienced transcriptional repression under chilling stress. Interestingly, up-regulated DEGs showed significant enrichment in pathways such as ‘Ribosome biogenesis (ko03009)’, ‘Thiamine metabolism (ko00730)’, ‘Ribosome biogenesis in eukaryotes (ko03008)’, ‘Enzymes with EC numbers (ko99980)’, ‘Plant hormone signal transduction (ko04075)’ together with ‘Transporters (ko02000)’ during the MLT phase. While in the ELT stage, ‘Thiamine metabolism (ko00730)’, ‘Sesquiterpenoid and triterpenoid biosynthesis (ko00909)’, ‘Transcription factors (ko03000)’, ‘MAPK signaling pathway-plant (ko04016)’, ‘Ubiquitin system (ko04121)’ as well as ‘Carotenoid biosynthesis (ko00906)’ pathways were significantly enriched. These findings implied that *P. catalpifolia* potted seedlings could activate the phytohormone signal transduction or MAPK (mitogen-activated protein kinases) signaling cascades, enhance the activity of transporters or transcription factors, along with modulate the vitamin or terpenoid metabolic processes to alleviate the damage caused by MLT or ELT.

### Functional enrichment analysis of gene datasets between different degrees of chilling stress

**Fig. 4:**
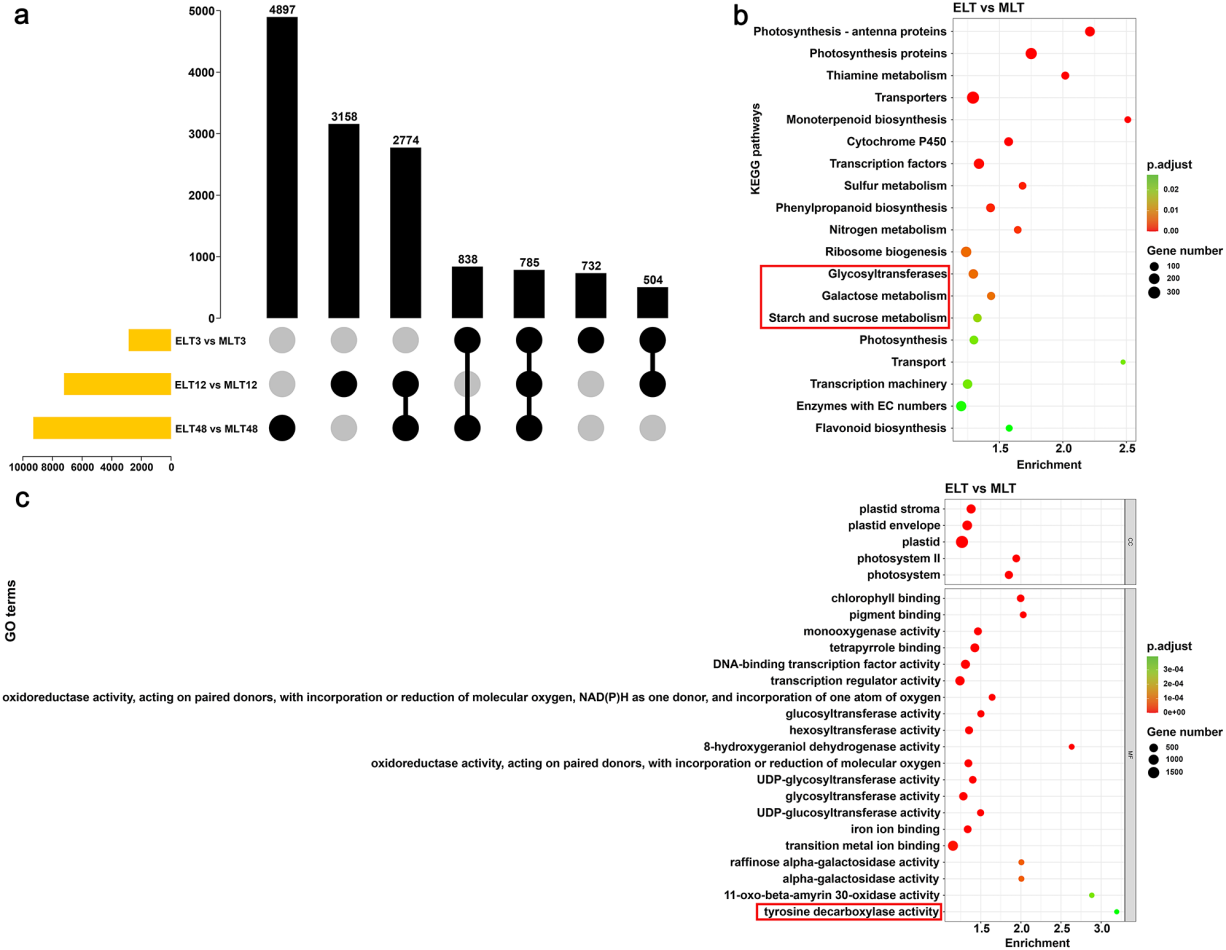
The upset plot among the ELT3 vs. MLT3, ELT12 vs. MLT12 and ELT48 vs. MLT48 comparative pairs (**a**), the left histogram displayed the DEG counts for each individual dataset. The solid black dots at the bottom indicated that there were intersections among the corresponding datasets. A bar chart at the top represented the number of intersecting elements within these converging datasets. The prominently enriched KEGG pathways (**b**) and GO terms (**c**) within the merged dataset of DEGs in ELT3 vs. MLT3, ELT12 vs. MLT12 and ELT48 vs. MLT48 were shown. GO term and KEGG pathways significantly associated with disparate contents of soluble protein and soluble sugar between MLT and ELT were marked with red boxes

The results based on a pairwise comparison between ELT and MLT at the same time intervals (ELT3 vs. MLT3, ELT12 vs. MLT12 and ELT48 vs. MLT48) revealed that the DEG counts gradually increased with the extension of stress time. A considerable rise in the number of DEGs was observed after 12 h cold exposure, and this increase would continue to 48 h, likely revealing a direct correlation between DEGs counts and the duration of stress. A total of 2859 (966 up-/1893 down-regulated), 7221 (4040 up-/3181 down-regulated) and 9294 (5330 up-/3964 down-regulated) DEGs were identified in ELT3 vs. MLT3, ELT12 vs. MLT12 and ELT48 vs. MLT48 comparative pairs, respectively (Additional file 2; Fig. 3b). To better understand the molecular mechanism resulting in variable soluble sugar and soluble protein contents between MLT and ELT, we performed a functional enrichment analysis on the combined DEGs (ELT vs. MLT) across 3 h, 12 h and 48 h, which could also provide support for further trend analysis. This conjoined dataset demonstrated a total of 13,688 DEGs (Fig. 4a). Furthermore, GO enrichment analysis revealed various significant terms, such as ‘plastid (GO: 0009536)’ and ‘photosystem (GO: 0009521)’ (CC), ‘chlorophyll binding (GO: 0016168)’, ‘monooxygenase activity (GO: 0004497)’, ‘DNA-binding transcription factor activity (GO: 0003700)’ and ‘pigment binding (GO: 0031409)’ (MF). At the same time, the 13,688 DEGs mainly concentrated within specific KEGG pathways, like ‘Photosynthesis-antenna proteins (ko00196)’, ‘Photosynthesis proteins (ko00194)’, ‘Thiamine metabolism (ko00730)’, ‘Transporters (ko02000)’ and ‘Monoterpenoid biosynthesis (ko00902)’ (Additional file 5). It indicated that the more significant expression alterations of DEGs linked to transcription factors, monooxygenase and transporters activities, secondary metabolites biosynthesis along with photosynthetic proteins in ELT compared to MLT.

It was noteworthy that the GO terms ‘tyrosine decarboxylase activity (GO: 0004837)’ and ‘aromatic-L-amino-acid decarboxylase activity (GO: 0004058)’, with potential associations to soluble proteins, were also notably enriched (Fig. 4c). By coomassie brilliant blue G-250 staining, the chromogenic substances of soluble protein were mainly made up of arginine and aromatic amino acids including tryptophan, tyrosine and phenylalanine. Tyrosine decarboxylase was known to catalyze the biosynthesis of specific metabolites derived from tyrosine. Our findings do not indicate any significantly enriched pathways related to arginine or other aromatic amino acid biosynthesis or metabolism. Besides, KEGG pathways related to soluble sugar, such as ‘Glycosyltransferases (ko01003)’, ‘Galactose metabolism (ko00052)’ and ‘Starch and sucrose metabolism (ko00500)’, were also significantly enriched (Fig. 4b). The soluble sugar compounds determined by the anthrone method, predominantly consisted of oligosaccharides and polysaccharides. Annotations of DEGs involved in the above pathways were principally related to oligosaccharides including galactinol, raffinose, maltose, glucose and fructose, as well as polysaccharides such as starch. In summary, these highlighted pathways were considered as key regulators in the physiological perturbation observed between MLT and ELT.

### Comparison of gene temporal expression trends under different chilling stresses

**Fig. 5:**
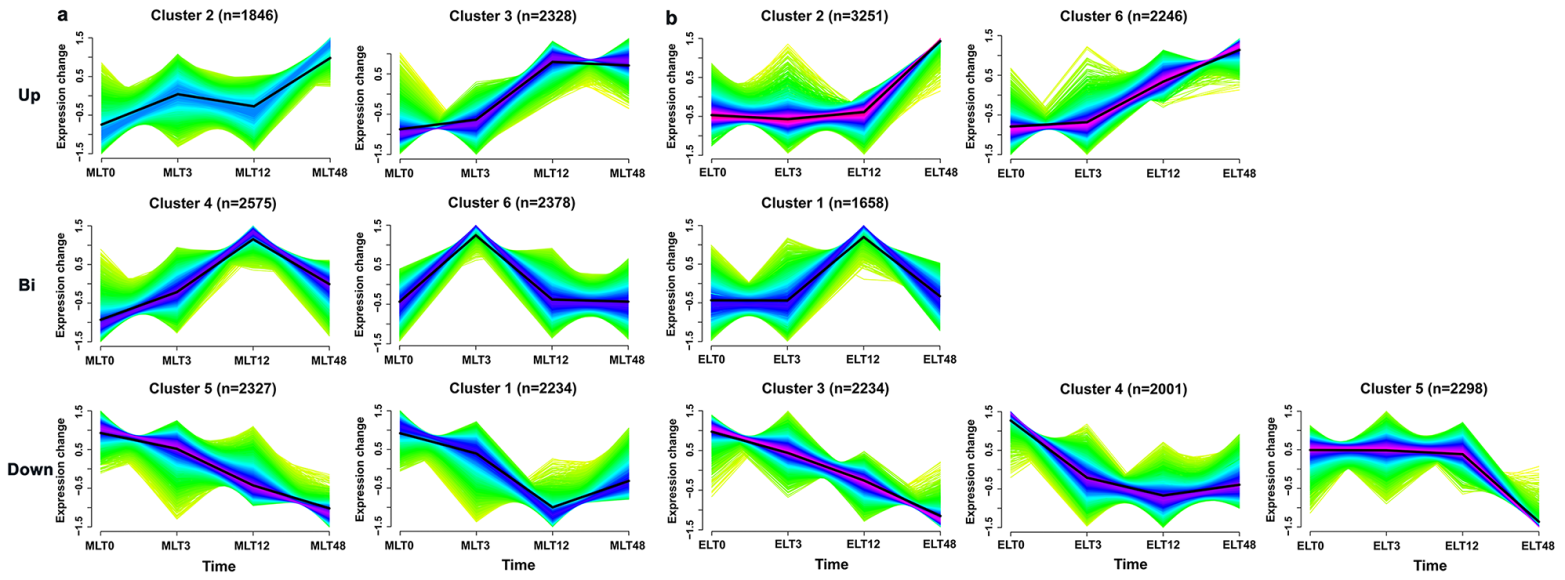
Expression trend analysis of 13,688 DEGs from ELT vs. MLT data sets. Profiles of MLT (**a**) and ELT (**b**) were classified into six, with the cluster numbers and DEGs counts displayed at the top of each profile, respectively. Profiles in the same row suggested parallel expression patterns between MLT and ELT. The three rows from top to bottom represented the expression trend of up-regulated (**Up**), biphasic-regulated (**Bi**) and down-regulated (**Down**), respectively. The y-axis denoted expression change, defined as the standardized value of log_2_ (FPKM + 1), while the x-axis represented different time nodes under diverse degrees of chilling stress

By employing the Mfuzz approach, 13,688 DEGs from ELT vs. MLT datasets, were categorized into six distinct clusters, so as to elucidate the expression patterns of DEGs between two degrees of chilling stress. Upon comparison with the 0 h, similar expression trends were found between clusters for both MLT (Fig. 5a) and ELT (Fig. 5b) on the same row. These trends included the up-regulation in clusters 2 and 3 of MLT along with clusters 2 and 6 of ELT, the down-regulation in clusters 1 and 5 of MLT as well as clusters 3, 4, and 5 of ELT, in addition to the biphasic expression patterns in clusters 4 and 6 of MLT together with cluster 1 of ELT, respectively.

According to the adjusted *P*-value, we found the most prominent GO term enrichment in MLT clusters 2 and 3 was ‘oligosaccharide catabolic process (GO: 0009313)’ and ‘cysteine-type peptidase activity (GO: 0008234)’. Simultaneously, ‘aromatic-L-amino-acid decarboxylase activity (GO: 0004058)’ and ‘heterocycle biosynthetic process (GO: 0018130)’ emerged as the main biological functions within ELT clusters 2 and 6. Furthermore, the ‘chloroplast stroma (GO: 0009570)’ and ‘cation binding (GO: 0043169)’ emerged as the most representative enriched terms in MLT clusters 1 and 5, while the ‘response to radiation (GO: 0009314)’, ‘thylakoid (GO: 0009579)’ and ‘lipid biosynthetic process (GO: 0008610)’ were observed to be considerably enriched within cluster 3, 4 and 5 of ELT, respectively. In the case of DEGs in MLT clusters 4 and 6, terms such as ‘cellular nitrogen compound metabolic process (GO: 0034641)’ and ‘nucleus (GO: 0005634)’ were considered to be the most representative, while ELT cluster 1 was the most enriched in ‘adenylyl-sulfate reductase (glutathione) activity (GO: 0033741)’ term (Additional file 5).

Meanwhile, KEGG enrichment analysis clarified distinctive metabolic pathways modulated by the DEGs across different clusters. In clusters 2 and 3, the DEGs in MLT were observed to enrich significantly the ‘Galactose metabolism (ko00052)’ and ‘Stilbenoid, diarylheptanoid and gingerol biosynthesis (ko00945)’ pathways, whereas DEGs in ELT were significantly enriched in ‘Ribosome biogenesis (ko03009)’ and ‘Thiamine metabolism (ko00730)’, emerged as main pathways affecting cluster 2 and cluster 6. For the DEGs in clusters 1 and 5 of MLT, the prominently enrichment of ‘Glyoxylate and dicarboxylate metabolism (ko00630)’ and ‘Photosynthesis (ko00195)’ pathways was discerned. While the ‘Photosynthesis proteins (ko00194)’ and ‘Monoterpenoid biosynthesis (ko00902)’ emerged as the primary pathways for the enrichment of DEGs in ELT clusters 3, 4 and 5. Additionally, ‘Thiamine metabolism (ko00730)’ and ‘Transcription factors (ko03000)’ pathways in MLT clusters 4 and 6, as well as ‘Sulfur metabolism (ko00920)’ pathway in ELT cluster 1 was identified as significantly enriched (Additional file 5). This analysis highlighted that most DEGs involved in the regulation of chilling stress response appeared to be associated with different metabolic pathways.

### Differential expression trend analysis of DEGs related to sugar metabolism

**Fig. 6:**
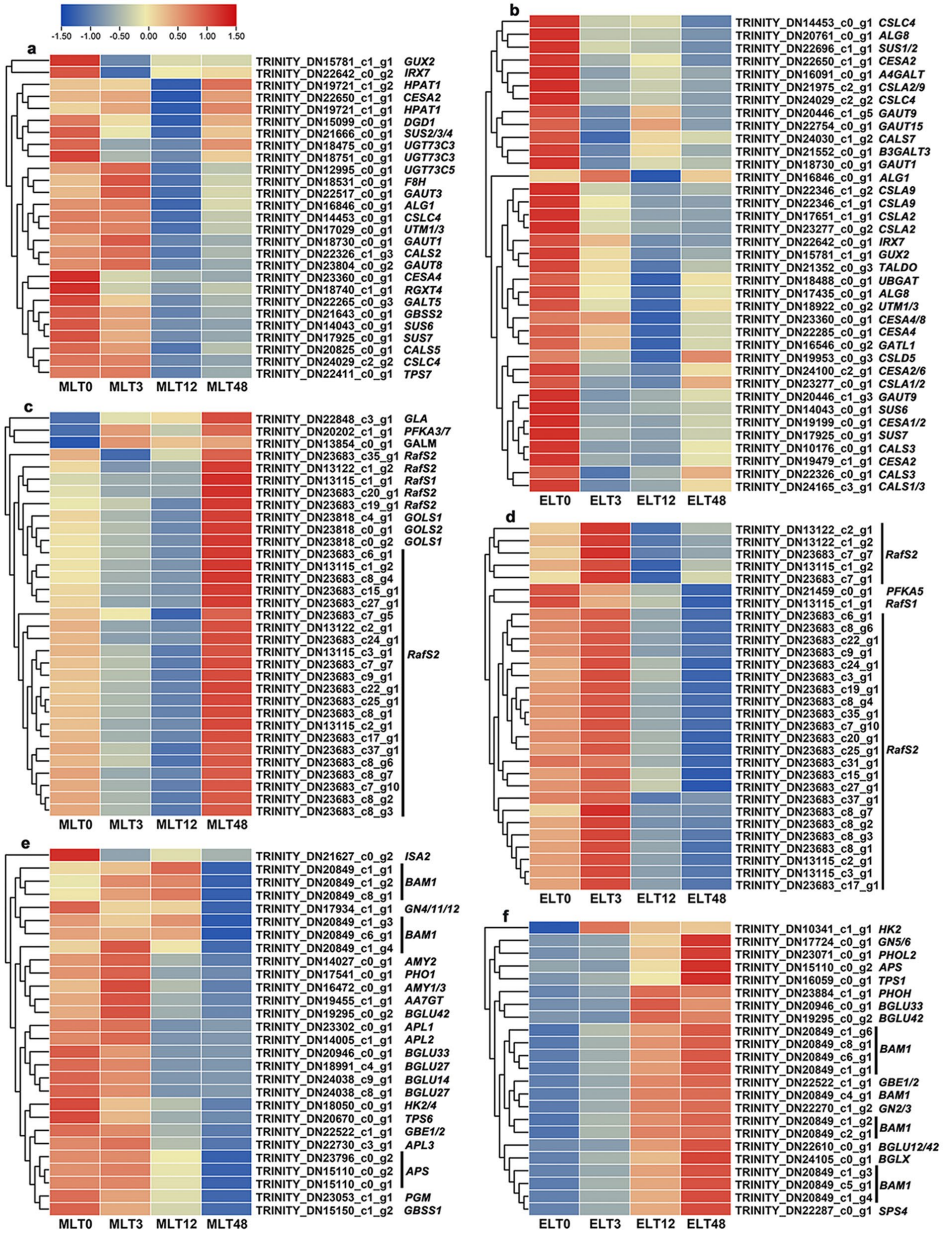
The heatmaps were visualized with standardized log_2_ (FPKM + 1) values, illustrated various expression trends for candidate DEGs participated in ‘Glycosyltransferases’ (**a**, **b**), ‘Galactose metabolism’ (**c**, **d**) and ‘Starch and sucrose metabolism’ (**e**, **f**) pathways between MLT (left side) and ELT (right side) in *P. catalpifolia*. Red and blue colors in the scale corresponded to up-regulated and down-regulated expression, respectively

Candidate genes displaying high expression abundance were primarily linked to the ‘Glycosyltransferases’, ‘Galactose metabolism’ and ‘Starch and sucrose metabolism’ pathways. Firstly, the DEGs in MLT cluster 1 (*n* = 27) and ELT cluster 4 (*n* = 37) demonstrated considerable enrichment in ‘Glycosyltransferases’ pathway. Compared with 0 h, an observable trend showed most *Galacturonosyltransferase* genes initially experiencing an up-regulation (3 h), followed by a down-regulation (12 h/48 h) in MLT (*GAUT1/3/8*). Conversely, they maintained a continuous downward trend (*GAUT1/9/15*) in ELT. Besides, most of the *cellulose synthase-like A/glucomannan 4-β-mannosyltransferase* genes were down-regulated in ELT (*CSLA2/9*), but not significantly enriched in MLT. Furthermore, the *UDP-glycosyltransferases TURAN* gene tended to be down-regulated (12 h/48 h) in MLT (*ALG1*), while in ELT (*ALG1*), it seemed to be constitutive expression (Fig. 6a and b). Secondly, the expression patterns of DEGs in the ‘Galactose metabolism’ pathway significantly varied between MLT cluster 2 (n = 33) and ELT cluster 3 (n = 30). Compared with 0 h, the expression of *galactinol synthase* (*GOLS1* and *GOLS2*) genes in MLT was primarily down-regulated (3 h/12 h) and then up-regulated (48 h), while no significant enrichment of these genes was observed in ELT. In addition, most *galactinol-sucrose galactosyltransferase 2*/*raffinose synthase 2* (*RafS2*) genes were firstly down-regulated (3 h/12 h) and then up-regulated (48 h) in MLT, while they were initially up-regulated (3 h) and then down-regulated (12 h/48 h) in ELT (Fig. 6c and 6d). Thirdly, DEGs participated in ‘Starch and sucrose metabolism’ pathway were assigned to MLT cluster 5 (n = 28) and ELT cluster 6 (n = 23). Compared with 0 h, most *β-amylase 1* (*BAM1*) genes were primarily up-regulated (3 h/12 h) and then down-regulated (48 h) in MLT, but in contrast, they were continuously up-regulated in ELT. Moreover, the expression levels of most *β-glucosidase* genes were down-regulated in MLT (*BGLU14/27/33/42*), but on the contrary, they were up-regulated in ELT (*BGLU33/42*) (Fig. 6e and 6f). In general, there were significant disparities in the expression trends of several DEGs involved in sugar metabolism between MLT and ELT, and some DEGs were uniquely enriched under specific low temperature conditions. These findings could provide molecular evidence for explaining the dissimilar soluble sugar content between diverse degrees of chilling stress.

### Differential expression trend analysis of DEGs associated with aromatic-L-amino-acid decarboxylase activity

**Fig. 7:**
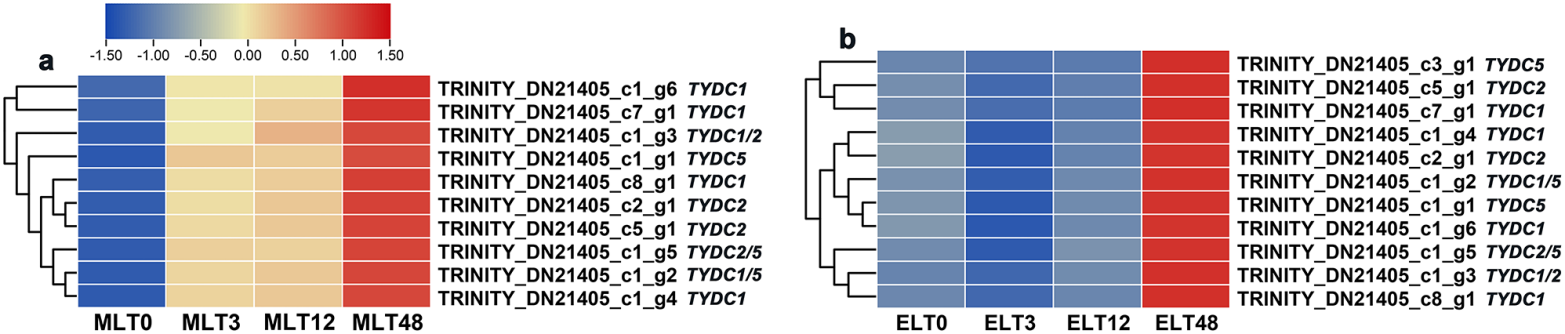
The normalized log_2_ (FPKM + 1) values enabled the visualization of heatmaps, representing the different expression trends of candidate DEGs engaged in ‘aromatic-L-amino-acid decarboxylase activity’ or ‘tyrosine decarboxylase activity’ term under MLT (**a**) and ELT (**b**) conditions within *P. catalpifolia*. Up-regulation and down-regulation of gene expression were described by the red and blue colors in the scale, respectively

In relation to ‘aromatic-L-amino-acid decarboxylase activity’, it was observed that only the *tyrosine/DOPA decarboxylase* (*TYDC*) genes were implicated. The expression levels of *TYDC1/2/5* genes illustrated a consistent up-regulation (cluster 2, *n* = 10) during MLT period (Fig. 7a). However, their expression levels exhibited a marked inhibition after 3 h and 12 h of ELT stress (cluster 2, *n* = 11), with a significant increase only observed after 48 h (Fig. 7b). Thus, the expression levels of *TYDC1/2/5* related to tyrosine metabolism in the short-term and medium-term of ELT were lower than those of MLT. These findings could provide a molecular basis for elucidating the accumulation of soluble proteins in ELT surpassed that in MLT.

### Expression verification analysis of DEGs

**Fig. 8:**
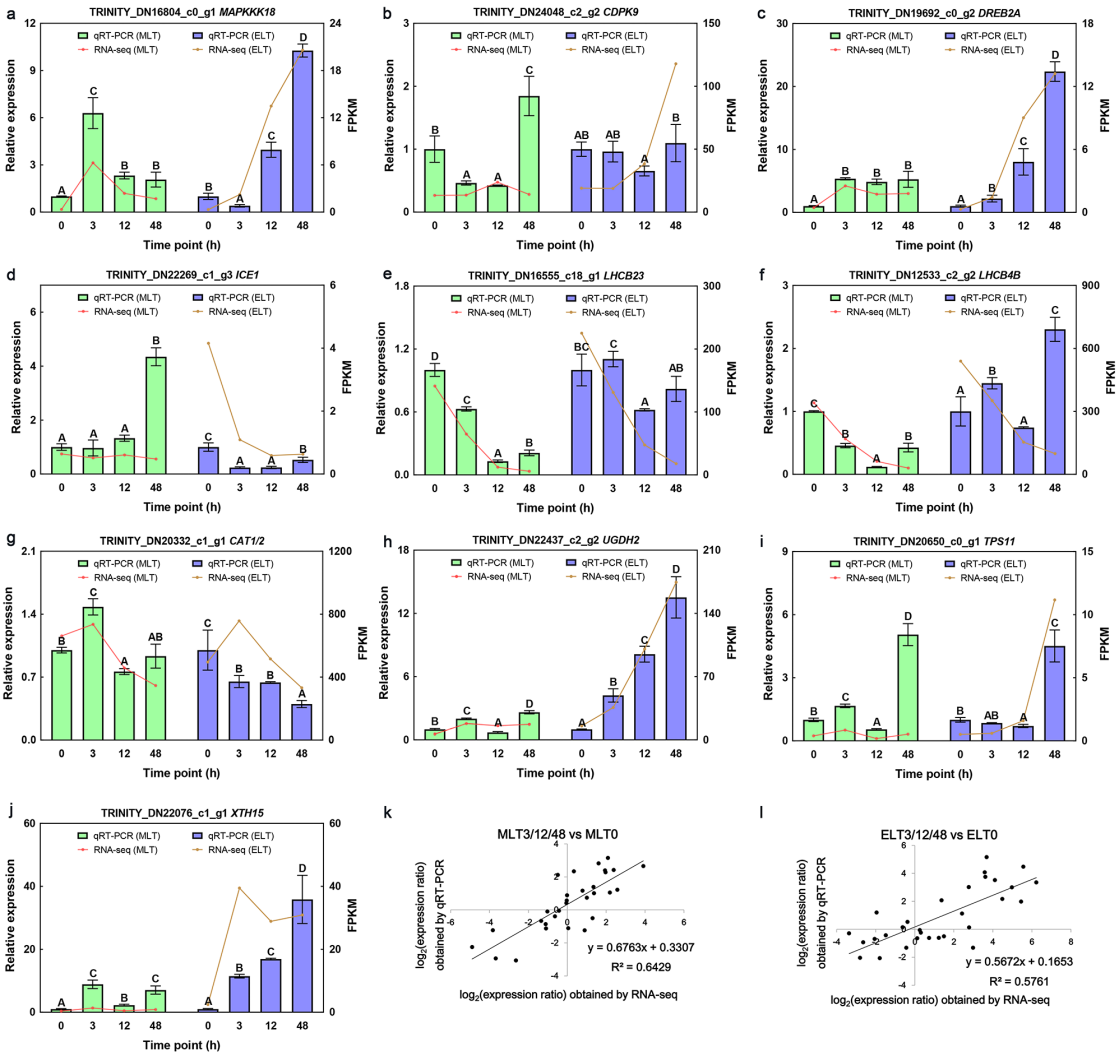
The reliability of RNA-seq data was validated by qRT-PCR analysis on ten candidate DEGs (**a-j**) in response to different degrees of chilling stress. The left y-axis depicted the relative expression levels determined by qRT-PCR, whereas the right y-axis showed the FPKM values from RNA-seq. The error value denoted the calculated mean ± standard deviation from three biological replicates, and different capital letters **A**-**D** indicated significant differences (Duncan, *P* < 0.01). **k**-**l** Linear regression analysis was performed on qRT-PCR (log_2_ FC) and RNA-seq data (log_2_ FC) of MLT3/12/48 vs. MLT0 and ELT3/12/48 vs. ELT0, respectively

The reliability of RNA-seq data was validated by conducting qRT-PCR analysis on ten randomly DEGs participated in low temperature response. *MAPKKK18* (Fig. 8a), *CDPK9* (Fig. 8b), *DREB2A* (Fig. 8c), *UGDH2* (Fig. 8h) and *TPS11* (Fig. 8i) genes were highly expressed during ELT period, while exhibited an initial up-regulation, followed by either down-regulation or remained stable during MLT phase. In the case of *ICE1* gene, an increased expression was recorded only after 48 h of MLT, while under ELT, it showed a decrease before increasing (Fig. 8d). The expression levels of *LHCB23* (Fig. 8e) and *LHCB4B* (Fig. 8f) demonstrated continuous decrease in both MLT and ELT treatments. Biphasic expression trends were noted for *CAT1/2* (Fig. 8g) and *XTH15* (Fig. 8j) in both MLT and ELT treatments, with transcript levels first increasing, and then decreasing. Moreover, the expression changes of *LHCB23/4B*, *UGDH2* and *XTH15* genes under ELT were more pronounced than those under MLT. According to linear regression analysis of qRT-PCR (log_2_ FC) and RNA-seq data (log_2_ FC), the correlation coefficients for MLT3/12/48 vs. MLT0 and ELT3/12/48 vs. ELT0 were 0.6429 (Fig. 8k) and 0.5761 (Fig. 8l), respectively. In general, the expression levels of the majority of candidate genes determined by qRT-PCR, were found to be in close agreement with the corresponding FPKM values from the RNA-Seq data.

## Discussion

### Physiological perturbation during chilling stress period

Plants often face the challenge of chilling or freezing temperatures in their natural habitats. Previous research had identified several plants such as *Spinacia oleracea* [18], *Arabidopsis thaliana* [19] and *Triticum aestivum* [20], demonstrated an increase in the accumulation of solutes including soluble sugars, free amino acids and vitamin C under suitable chilling stress. The accumulation of osmoregulation substances could effectively prevent the damage of low temperature by modulating the osmotic potential and reducing water loss [21]. Among them, sugars and amino acids were vital for plants to respond to chilling stress and played a dual role. They were not only essential for the synthesis of functional proteins, but also acted as precursor substances for a large number of multi-functional metabolites [9]. An investigation on *Prunus mume* showed that the contents of soluble sugar, soluble protein, and proline, as well as the activity of peroxidase (POD) increased in different degrees under various durations (0 d, 1 d, 3 d, 5 d, 7 d, 9 d and 11 d) of 4℃ cold acclimation [22]. Similar to these results, our study found an identical trend with a substantial increase in the concentrations of proline, soluble sugar and soluble protein, coupled with an enhanced activity of the antioxidant enzymes (SOD and CAT) during MLT and ELT periods. Interestingly, the fold changes in soluble sugar and soluble protein content appeared to be affected by the degree and duration of stress, indicating that long-term ELT might have a stronger physiological response than MLT. The accumulation of these osmoregulation substances seemed to enhance the chilling tolerance of plants, despite visible wilting symptoms were observed after long-term ELT exposure. Compared with MLT, a probable over-accumulation of soluble sugar in long-term ELT, might elevate the leaf osmotic potential and lead to physiological dehydration.

### Important terms or pathways enriched under different degrees of chilling stress

The functional analysis of DEGs in six comparative pairs (compared with 0 h) clarified that, a multitude of terms or pathways, including not only those correlating with primary metabolites, but also those related to secondary metabolites, were significantly enriched under MLT and ELT. For instance, several DEGs involved in pathways associated with sugar metabolism demonstrated down-regulated in MLT (e.g. ‘Galactose metabolism’ and ‘Glycolysis/Gluconeogenesis’) and ELT (such as ‘Glycosyltransferases’ and ‘Pentose and glucuronate interconversion’), while some other DEGs participated in terms including ‘raffinose family oligosaccharide biosynthetic process’ and ‘carbohydrate storage’, were up-regulated particularly in ELT. A marked up regulation was also observed in DEGs linked to aromatic amino acid metabolism, such as ‘tyrosine decarboxylase activity’ term during MLT. Importantly, during ELT period, a specific enrichment was found in secondary metabolic pathways such as ‘Cutin, suberine and wax biosynthesis (ko00073)’ and ‘Carotenoid biosynthesis (ko00906)’, in which DEGs concerned with the former were down-regulated while DEGs involved in the latter were up-regulated. Consequently, compared with MLT, the seedlings of *P. catalpifolia* might alleviate the adverse effects of ELT by enhancing the additional biosynthesis of secondary metabolites, which was similar to the defensive strategy of secondary metabolite accumulation in *Sophora alopecuroides* under various drought stress conditions. Integrative analysis of transcriptome and metabolome of *S. alopecuroides* clearly highlighted the key role of flavonoid metabolism, particularly the isoflavonoid biosynthesis pathway in drought tolerance [23].

Upon comprehensive analysis of significance and abundance, a total of 13,688 DEGs from ELT vs MLT datasets made a significant contribution to pathways including ‘Photosynthesis proteins’, ‘Photosynthesis-antenna proteins’, ‘Thiamine metabolism’, ‘Transporters’ and ‘Monoterpenoid biosynthesis’. It indicated that *P. catalpifolia* might respond to the strengthening of chilling stress by modulating the expression levels of DEGs in these pathways. Moreover, the enrichment of sugar-related pathways (‘Glycosyltransferases’, ‘Galactose metabolism’ and ‘Starch and sucrose metabolism’) and aromatic-L-amino-acid-related term (‘tyrosine decarboxylase activity’) was also evident. These expression differences could provide a molecular basis for the differential soluble sugar/protein content observed under distinct chilling stress conditions. Similarly, a previous study on *Camellia oleifera* flower buds reported an increase in D-fructose, inositol, glucose and sucrose concentrations with the duration of chilling stress treatment [7]. Transcriptome sequencing revealed numerous DEGs involved in cold acclimation, among which, the gene datasets associated with sugar metabolic enzymes and sugar transmembrane transporters emerged as significant functional gene clusters, revealing the transcriptional regulation mechanism of honey-like mucous substances formed at the base of flowers and around ovaries, which provided valuable genetic reserves for cold-tolerant breeding of *C. oleifera*.

### The expression trend of DEGs related to sugar and aromatic-L-amino-acid metabolism under different degrees of chilling stress

Several candidate DEGs associated with sugar metabolism were identified, mainly participating in ‘Glycosyltransferases,’ ‘Galactose metabolism,’ and ‘Starch and sucrose metabolism’ pathways. We observed differential expression patterns of these DEGs between MLT and ELT. Particularly, nine DEGs implicated in the ‘Glycosyltransferases’ pathway, including *GAUT* and *CESA* genes, were co-expressed under both MLT and ELT stress conditions (Additional file 1: Fig. S3a). Upon exposure to 4℃ for various durations (1 d, 30 d, 60 d, 90 d and 150 d), *GAUT6* and *GAUT14* genes involved in cell wall and pectin biosynthesis, were identified in apple fruits. The expression levels of these genes significantly decreased during short-term cold storage (30 d/60 d) and increased during long-term cold storage (90 d/150 d) [24]. The results of this research, indicating a downward trend with the *GAUT* genes in both MLT (*GAUT1/3/8*) and ELT (*GAUT1/9/15*), were slightly different from the previous findings. It might be related to the differences in stress duration and tissue location. Furthermore, the transcript levels of *CESA2* and *CESA6* genes, regulating cellulose synthesis in *Rosa xanthina*, were markedly down-regulated following exposure to 4℃ [25]. Similar findings were observed in the present study. The *CESA* genes in MLT (*CESA2/4*) and ELT (*CESA1/2/4/6*) showed a downward trend. Interestingly, several genes, such as *UGT73C3/C5* in MLT and *CSLA2/9* in ELT, were uniquely enriched at specific low temperatures, exhibiting a downward trend. Previous research had shown that the *UGT73C5/C6* gene in *A. thaliana* could catalyze the 23-O glycosylation of brassinosteroids (BRs), subsequently leading to their inactivation [26]. During MLT period, the down-regulation of *UGT73C3/5* homologous genes expression in *P. catalpifolia* might inhibit BR inactivation and enhance cold tolerance. Moreover, compared with 0 h, the expression levels of *CsCLS2/15/34* genes in *Cucumis sativus* were significantly decreased after cold treatment for 24 h [27], which were consistent with the down-regulation of the *CSLA2/9* homologous genes observed during ELT period in this study.

Several DEGs participating in ‘Galactose metabolism’ pathway, were identified. 26 of them intersected between MLT and ELT, mainly including *RafS1/2* genes (Additional file 1: Fig. S3b). During MLT period, the expression pattern of *RafS2* genes initially decreased at 3 h, then reached a minimum at 12 h, and finally returned to a peak at 48 h. While in ELT, the transcript levels of *RafS2* genes began to increase after 3 h, then reduced after 12 h, and lastly declined to the lowest after 48 h. Raffinose family oligosaccharides (RFOs), were considered as dehydration protectants [28], for their important effects on plant abiotic stress tolerance. These RFOs were synthesized with galactose and sucrose as substrates under the action of raffinose synthase (galactinol-sucrose galactosyltransferase) [29]. Co-overexpression of *ZmGOLS2* and *ZmRafS* or singular overexpression of *ZmGOLS2* triggered a significant increase in RFOs content of *A. thaliana* seedlings. Interestingly, overexpression of *ZmRafS* increased raffinose content but reduced the concentration of galactose, stachyose and verbascose [30]. Three *GOLS* genes (two *GOLS1* and one *GOLS2*), identified in this study, were significantly enriched in MLT. Their expression patterns were similar to those of *RafS2* genes, showing a downward trend under short-term and medium-term chilling stresses. After long-term chilling stress, the transcript levels of both *GOLS* and *RafS2* were increased. However, changes in metabolite content lagged behind the rapid transcript fluctuations. Although the soluble sugar content of MLT48 h increased obviously compared to 0 h, it still remained lower than that of ELT48 h. Nevertheless, the *GOLS* gene was not significantly enriched in ELT. Based on the continuous down-regulation of *RafS2* gene throughout medium-to-long term ELT, it could be inferred that the sustained accumulation of soluble sugars (except raffinose) like galactose, stachyose and verbascose was evidenced, revealing the possible molecular mechanism of soluble sugar content changes.

Approximately ten DEGs involved in ‘Starch and sucrose metabolism’ pathway, exhibited co-expression between MLT and ELT, such as *BAM1* and *BGLU* genes (Additional file 1: Fig. S3c). Upon exposing *Vitis vinifera* seedlings to cold environments, the transcript abundance of *VvBAM1* gene was significantly increased. Ectopic overexpression of *VvBAM1* gene in tomato could enhance amylase activity, affect the contents of soluble sugars such as glucose and sucrose in transgenic plants, promote reactive oxygen scavenging, thereby improving cold tolerance [31]. Besides, a remarkable rise in the transcript level of *BGLU* gene from chickpea was triggered after exposure to cold hardening at 10℃ for 5 d [32]. Furthermore, a considerable upregulation in the expression levels of *BGLU12* and *BAM1* genes corresponding to the regulation of glucose and maltose synthesis was detected in *Rosa xanthina* following 4℃ treatment [25]. The above findings were consistent with the results of this study. Multiple *BAM1* homologous genes experienced an up-regulated expression trend in the short-term and medium-term of MLT treatment, but decreased to the lowest expression level in the long-term of MLT treatment. On the contrary, these genes consistently showed up-regulation during ELT period. Furthermore, most of the *BGLU* genes were continuously down-regulated during MLT phase but maintained their up-regulation during ELT phase. Hence, long-term ELT exposure could lead to the higher expression levels of *BAM* and *BGLU* as compared to long-term MLT exposure. These two genes induced the synthesis of glucose and maltose, contributing towards higher soluble sugar accumulation in ELT. To sum up, the sugar signals stimulated by *BAM1* and *BGLU* genes might be pivotal in modulating the chilling tolerance of *P. catalpifolia*.

L-tyrosine decarboxylase (TYDC) mediated the decarboxylation of L-tyrosine to produce tyramine and dopamine [33], which was a key step in secondary metabolic reactions of higher plants. It was the initial enzyme connecting primary metabolism and secondary metabolism [34]. So far, the characteristics of *TYDC* genes had been reported in various plant species [35–37]. In this study, an overlap of ten DEGs linked to ‘tyrosine decarboxylase activity’ was found between MLT and ELT (Additional file 1: Fig. S3d). The expression levels of *TYDC1/2/5* genes remained increasing during MLT period. In contrast, during ELT phase, most of them demonstrated a down-regulation at 3 h and 12 h, but then exhibited an up-regulation that peaked at 48 h. Such patterns indicated the consumption of L-tyrosine continued to increase during MLT treatment, while decreased in the short-term and medium-term of ELT treatment. This might explain the higher soluble protein content observed in ELT than in MLT.

## Conclusions

Based on the integrated analysis of physiological characteristics and comparative transcriptome in potted seedlings of *P. catalpifolia* under different degrees of chilling stress, this investigation explored the fluctuation patterns of osmoregulation substance (such as soluble sugar and soluble protein) contents, as well as their probable genetic foundation. Interestingly, extreme low temperature conditions significantly elevated the contents of soluble sugar and soluble protein compared to moderate low temperature conditions. A range of potential DEGs enriched in ‘Glycosyltransferases’, ‘Galactose metabolism’ and ‘Starch and sucrose metabolism’ pathways, as well as ‘tyrosine decarboxylase activity’ term had been distinguished. Certain genes, including *cellulose synthase-like A* (*CLSA2/9*), *raffinose synthase* (*RafS1/2*), *β-amylase* (*BAM1*) and *tyrosine/DOPA decarboxylase* (*TYDC1/2/5*), displayed differential expression trends under two degrees of chilling stress, implying their essential role in regulating the accumulation of osmoregulation substances during chilling stress periods. In general, although there were several osmo-regulative genes with differential expression patterns, *P. catalpifolia* might counteract diverse degrees of chilling stress through partially overlapping metabolic pathways. This study can offer valuable physiological and molecular insights into how *P. catalpifolia* adapts to challenging complex low-temperature environments.

### Methods

#### Plant materials and chilling stress treatment

Root segments from *P. catalpifolia* were supplied by the germplasm innovation institute of Shandong provincial forest and grass germplasm resource center (Jinan, China). Asexual lineages generated through root induction methods were subjected to preliminary screening using natural winter chilling stress (> 0℃). Dormant branches which had experienced cold acclimation were then pruned, and their sprouting buds were harvested in the following spring, serving as materials for rapid in vitro propagation. Then the primary culture (MS + 3 mg L^-1^ 6-BA + 0.3 mg L^-1^ NAA + 3% sucrose + 0.8% agar) and subculture (MS + 2 mg L^-1^ 6-BA + 0.3 mg L^-1^ NAA + 3% sucrose + 0.8% agar) were carried out on a series of mediums. The ambient temperature was maintained at 25 ± 2℃, with alternating 12 h light and dark periods. Seedlings sub-cultured for 40 d were then used for rooting culture (1/2 MS + 0.1 mg L^-1^ NAA + 2% sucrose + 0.3% phytagel). Following a rooting period of 20 d, plantlets were transplanted into a mixed matrix (peat soil: vermiculite: perlite = 1: 1: 1) for an additional 40 d (Additional file 1: Fig. S1). Plantlets displaying uniform growth in the chamber (25℃/23℃, 14 h/10 h) were exposed to moderate low temperature (15℃) and extreme low temperature (5℃) treatments for 0 h, 3 h, 12 h and 48 h, as well as ambient temperature (25℃) recovery for 2 d, respectively, collecting leaves and photographing seedlings at above intervals for physiological evaluation and phenotypic observation. Besides, the samples were collected at 0 h, 3 h, 12 h and 48 h for RNA-seq and quantitative real-time PCR (qRT-PCR) analysis during MLT and ELT periods. Each biological replicate consisted of leaves from four individual plants, with a total of three replicates obtained. All samples were immediately frozen in liquid nitrogen and stored at -80℃.

### Determination of physiological indices

The relative electrolyte leakage (%) was measured by HI98303 EC tester following the procedure detailed by Peng et al. [38], and the soluble protein content (mg g^-1^ FW, 595 nm) was estimated based on the method of Bradford [39]. Bovine serum albumin (Macklin, Shanghai, China) was employed as a reference standard. Activity assays for SOD (U mg^-1^ prot, 560 nm) and CAT (U mg^-1^ prot, 240 nm), as well as concentration measurements of soluble sugar (mg g^-1^ FW, 620 nm) and proline (µg g^-1^ FW, 520 nm) were conducted with detection kits (Genepioneer, Nanjing, China). All measurements were in accordance with the microplate method instructions provided with the kits. Absorbance measurements were performed at the referenced wavelengths by SuPerMax series multimode plate reader (Flash, Shanghai, China). Data were processed and transformed into histograms via GraphPad Prism 7 software (GraphPad, La Jolla, CA), with statistics expressed as mean ± standard deviation.

### RNA extraction, cDNA library construction and sequencing

Total RNA extraction reagent (Trizol) kit was used to isolate RNA from samples collected at different time intervals (0 h, 3 h, 12 h and 48 h) in MLT and ELT. Evaluation of the purity (total content > 7 µg, A260/280 ratio > 1.9 and A260/230 ratio > 1.7), concentration (> 200 ng µL^-1^) and structural integrity (RIN value > 8.3) of RNA was performed by agarose gel electrophoresis, NanoPhotometer spectrophotometer and Agilent 2100 bioanalyzer, respectively. Samples meeting these standards underwent DNase I digestion followed by transcriptome sequencing. The cDNA libraries were constructed according to the protocols provided by Illumina manufacturer. Finally, sequencing was conducted by igenebook biotechnology company (Wuhan, China) on the Illumina HiSeqTM 2000 sequencing platform, with a paired-end (PE150) sequencing approach.

### Assembly, annotation and expression quantitative analysis

In our previously unpublished study, we collected RNA-seq data from 12 distinctive samples (NT, 25℃ for 0, 3, 12 and 48 h). Complimenting these results, the current investigation further contributed an additional set of RNA-seq data from 24 samples (MLT/ELT, 15℃/5℃ for 0, 3, 12 and 48 h). This combined dataset consisting of 36 samples was employed for the assembly of a reference transcriptome. The Cutadapt software [40] performed data filtration, and FastQC software [41] was used to ensure data quality. Detailed statistics on both raw and clean data yielded fundamental data attributes. Due to the absence of a reference genome for *P. catalpifolia*, all the clean data was subjected to *de novo* assembly using Trinity software [42], yielding contigs and unigenes. BlastX was utilized to annotate these unigenes against Nr, UniProt, GO and KEGG databases (E-value < e^-5^). Bowtie2 software [43], was utilized to map the cleaned data to the *de novo* assemblies. The abundance of assembled transcripts and the expression level of unigenes were estimated with RSEM (RNA-Seq by Expectation-Maximization) software, and standardized as FPKM to normalize for gene length and sequencing depth. According to the gene expression data of 24 samples in this study, the Pearson correlation coefficients were calculated to illustrate the correlations between samples, which were then visualized using a heat map. Moreover, principal component analysis (PCA) was conducted using online bioinformatics tools (https://www.bioinformatics.com.cn/plot_basic_PCA_plot_034).

### Differential expression analysis

The DESeq2 software [44] was employed to examine the significant differences in gene expression. First, to meet the hypothesis of the linear model, the expression levels from each sample were initially normalized and log_2_ converted. Following that, the false discovery rate (FDR) was calculated using the BH method. Next, a linear model was employed to evaluate the variations in gene expression at 0 h, 3 h, 12 h and 48 h, including MLT3 vs. MLT0, MLT12 vs. MLT0, MLT48 vs. MLT0, ELT3 vs. ELT0, ELT12 vs. ELT0 and ELT48 vs. ELT0, as well as ELT3 vs. MLT3, ELT12 vs. MLT12, ELT48 vs. MLT48. The screening thresholds of DEGs were established as FDR ≤ 0.05, and |log_2_ FC|≥1. The number of DEGs in the short-, medium- and long-term responses to MLT and ELT was determined in the nine comparative pairs and visualized as a volcano plot, respectively.

### GO and KEGG analysis

Small tools provided within the igenebook cloud platform, as well as TBtools software [45] were employed for GO and KEGG enrichment analysis (adjusted *P*-value ≤ 0.05) of DEGs across nine comparative pairs, as well as assigned gene sets (including time series clusters), respectively. From the 13,688 DEGs in the merged ELT vs. MLT dataset, we identified the top 25 significantly enriched terms and pathways, as demonstrated in the bubble diagrams. In addition, comprehensive details were available in Additional files 2 and 5.

### Upset plots and Venn diagrams

For the dataset of DEGs in ELT vs. MLT over all time intervals, gene counts were further analyzed using upset plots created by TBtools software. Additionally, Venn diagrams were generated to visually represent the number of both shared and unique DEGs associated with sugar and aromatic amino acid metabolism under two distinct degrees of chilling stress (Additional file 1: Fig. S3).

#### Time series gene clustering analysis

The Mfuzz online tool [46] helped to define the temporal traits of 13,688 DEGs in the ELT vs. MLT merged datasets, and further categorize them into six distinctive profiles in MLT and ELT, respectively. For each profile, several enriched GO terms or KEGG pathways that showed potential linkage to aromatic L-amino acid decarboxylase activity and sugar metabolism were identified. Ultimately, we used the normalized log_2_ (FPKM + 1) values generated by TBtools to visualize the expression data as a heat map (Addition file 6).

### Verification of RNA-seq data by qRT-PCR

The leaves of *P. catalpifolia* were harvested for total RNA extraction. With 1 µg of total RNA as the template, the first strand of cDNA was synthesized by Takara PrimeScript RT kit. The qRT-PCR reaction was performed on the Line Gene 9600 fluorescence quantitative PCR detection system (FQD-96 A, Bori Technology, Hangzhou, China) with the application of Tb Green^®^ Premix Ex Taq TM II (Takara, Beijing, China). The total volume for the PCR reaction was 20 µL, comprising of 10 µL SYBR Green PCR mix, 1 µL cDNA, 0.8 µL of both forward and reverse primers, 0.4 µL ROX reference dye II and 7.0 µL ddH_2_O. The PCR reaction followed this procedure: 95℃ 2 min, 95℃ 5 s, 60℃ 34 s, 40 cycles. The *TUB5* gene of *P. fortunei* was used as the internal reference [47]. The expression level of the samples exposed to low temperature for 0 h was set to 1, with the relative expression level standardized by 2^-ΔΔCt^ method. The expression levels of ten DEGs, including *MAPKKK18* (TRINITY_DN16804_c0_g1), *CDPK9* (TRINITY_DN24048_c2_g2), *DREB2A* (TRINITY_DN19692_c0_g2), *ICE1* (TRINITY_DN22269_c1_g3), *LHCB23* (TRINITY_DN16555_c18_g1), *LHCB4B* (TRINITY_DN12533_c2_g2), *CAT1/2* (TRINITY_DN20332_c1_g1), *UGDH2* (TRINITY_DN22437_c2_g2), *TPS11* (TRINITY_DN20650_c0_g1) and *XTH15* (TRINITY_DN22076_c1_g1) were detected by qRT-PCR. Three individual plants were combined for each biological repeat, and there were three biological repeats. The histograms were converted by GraphPad Prism 7 software with mean ± standard deviation applied. Primers for expression analysis were designed in the non-conservative region of the above genes (Additional file 1: Table S3). The correlation assessment between the relative expression value (log_2_ FC) from qRT-PCR and the FPKM value (log_2_ FC) from RNA-seq was evaluated.

### Statistical analysis

The differences among the samples were assessed by the application of a one-way ANOVA. All analyses were conducted by SAS. The statistical significance of the difference was determined using Duncan’s multiple range test at a *P*-value < 0.01.

### Electronic supplementary material

Below is the link to the electronic supplementary material.

Supplementary Material 1

Supplementary Material 2

Supplementary Material 3

Supplementary Material 4

Supplementary Material 5

Supplementary Material 6

## Data Availability

The raw sequencing data supporting the current study is deposited in NCBI, with accession number PRJNA1004893 (https://www.ncbi.nlm.nih.gov/sra/PRJNA1004893).

## Abbreviations

6-BA: N-(Phenylmethyl)-9 H-purin-6-amine
AP2/ERF: APETALA2/ethylene responsive transcription factor
AREB/ABF: ABA responsive element binding protein/ABRE binding factors
bHLH: basic helix-loop-helix
bZIP: basic leucine zipper
cDNA: complementary DNA
DREB1/CBF: Dehydration responsive element binding 1/C-repeat binding factor
FC: Fold change
HSF: Heat shock transcription factor
MS: Murashige and Skoog medium
NAA: α-naphthaleneacetic acid
PCR: Polymerase chain reaction
RIN: RNA integrity number
qRT-PCR: Quantitative real-time PCR
ZFP: Zinc finger proteins
