# Supplementary material for: Comparative transcriptomic profiles of Paulownia catalpifolia under different degrees of chilling stress during the seedling stage
Source: BMC Genomics. 2024 Jul 24;25:716. doi: 10.1186/s12864-024-10613-7 (PMC11270786; doi:10.1186/s12864-024-10613-7)
Supplement: Supplementary file 2 — Supplementary Material 2 [file 12864_2024_10613_MOESM2_ESM.doc]

**
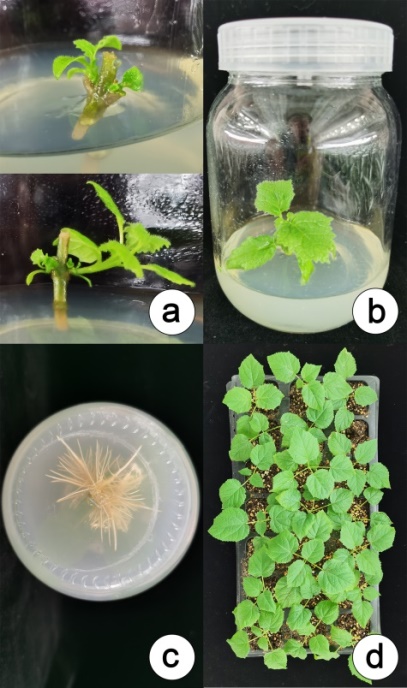
**

**Fig. S1** Establishment of rapid in vitro propagation system of *Paulownia catalpifolia*. **a** Primary culture; **b** Sub-culture; **c** Rooting culture; **d** Transplanting

**Table S1** Assembly results statistics

| Item | Count |
| --- | --- |
| Total trinity genes | 106759 |
| Total trinity transcripts | 222910 |
| Total assembled bases | 79874648 |
| Median contig length | 340 |
| Average contig | 748.18 |
| Contig N50 | 1581 |

**Note**: Total trinity genes/transcripts: The number of trinity genes/transcripts sequences obtained by assembly; Total assembled bases: The number of bases of total trinity genes/transcripts assembled; Median contig length: The contig length in the middle of the sequence, which was formed by arranging the length of contigs in order of size; Average contig: The average length (bp) of all contigs obtained by assembly; Contig N50: According to the length, the assembled contigs are sorted from large to small, and the corresponding contig length is accumulated to half of the total length.

**Table S2** Functional annotation of unigenes statistics

| Item | Count (ratio) |
| --- | --- |
| Unigene number | 106759 |
| GO | 34940 (32.73%) |
| Uniprot | 34697 (32.50%) |
| Nr | 25257 (23.66%) |
| Pfam | 18040 (16.90%) |
| KEGG | 17975 (16.84%) |
| At least one hit by BLASTX | 39949 (37.42%) |

**Note**: The number of unigenes matched with GO, UniProt, Nr, Pfam and KEGG databases, respectively

**Table S3** Mapping statistical table with assembly results

| Sample_id | Clean reads | Mapped reads | Mapped ratio |
| --- | --- | --- | --- |
| MLT0_1 | 41382128 | 28424440 | 68.69% |
| MLT0_2 | 40268588 | 26810756 | 66.58% |
| MLT0_3 | 37324250 | 26083134 | 69.88% |
| MLT3_1 | 42933820 | 30850908 | 71.86% |
| MLT3_2 | 40478574 | 26227344 | 64.79% |
| MLT3_3 | 41469980 | 27143748 | 65.45% |
| MLT12_1 | 44994960 | 29784642 | 66.20% |
| MLT12_2 | 41626246 | 27561400 | 66.21% |
| MLT12_3 | 44791940 | 29465530 | 65.78% |
| MLT48_1 | 46864778 | 30898520 | 65.93% |
| MLT48_2 | 44643996 | 29509788 | 66.10% |
| MLT48_3 | 42073472 | 27972518 | 66.48% |
| ELT0_1 | 37521108 | 26069664 | 69.48% |
| ELT0_2 | 41212560 | 27568154 | 66.89% |
| ELT0_3 | 43106176 | 29023310 | 67.33% |
| ELT3_1 | 40185936 | 27889830 | 69.40% |
| ELT3_2 | 38137014 | 25818782 | 67.70% |
| ELT3_3 | 36107470 | 24288544 | 67.27% |
| ELT12_1 | 40572936 | 28140756 | 69.36% |
| ELT12_2 | 40910106 | 28410832 | 69.45% |
| ELT12_3 | 37607622 | 26296520 | 69.92% |
| ELT48_1 | 38112108 | 25795542 | 67.68% |
| ELT48_2 | 43798726 | 29739416 | 67.90% |
| ELT48_3 | 37892102 | 26371926 | 69.60% |

Note: MLT represented moderate low temperature (15℃), ELT represented extreme low temperature (5℃); The RNA-seq samples of MLT/ELT were designated as MLT/ELT0_1/2/3, MLT/ELT3_1/2/3, MLT/ELT12_1/2/3 and MLT/ELT48_1/2/3, respectively (0 represented the control, 3, 12 and 48 represented treatment duration of 3 h, 12 h and 48 h, _1/2/3 represented three independent biological replicates); Clean reads: Number of filtered data; Mapped reads: The number of clean reads that can be aligned to the assembled transcripts; Mapped ratio: The percentage of clean reads mapped to the assembled transcripts.


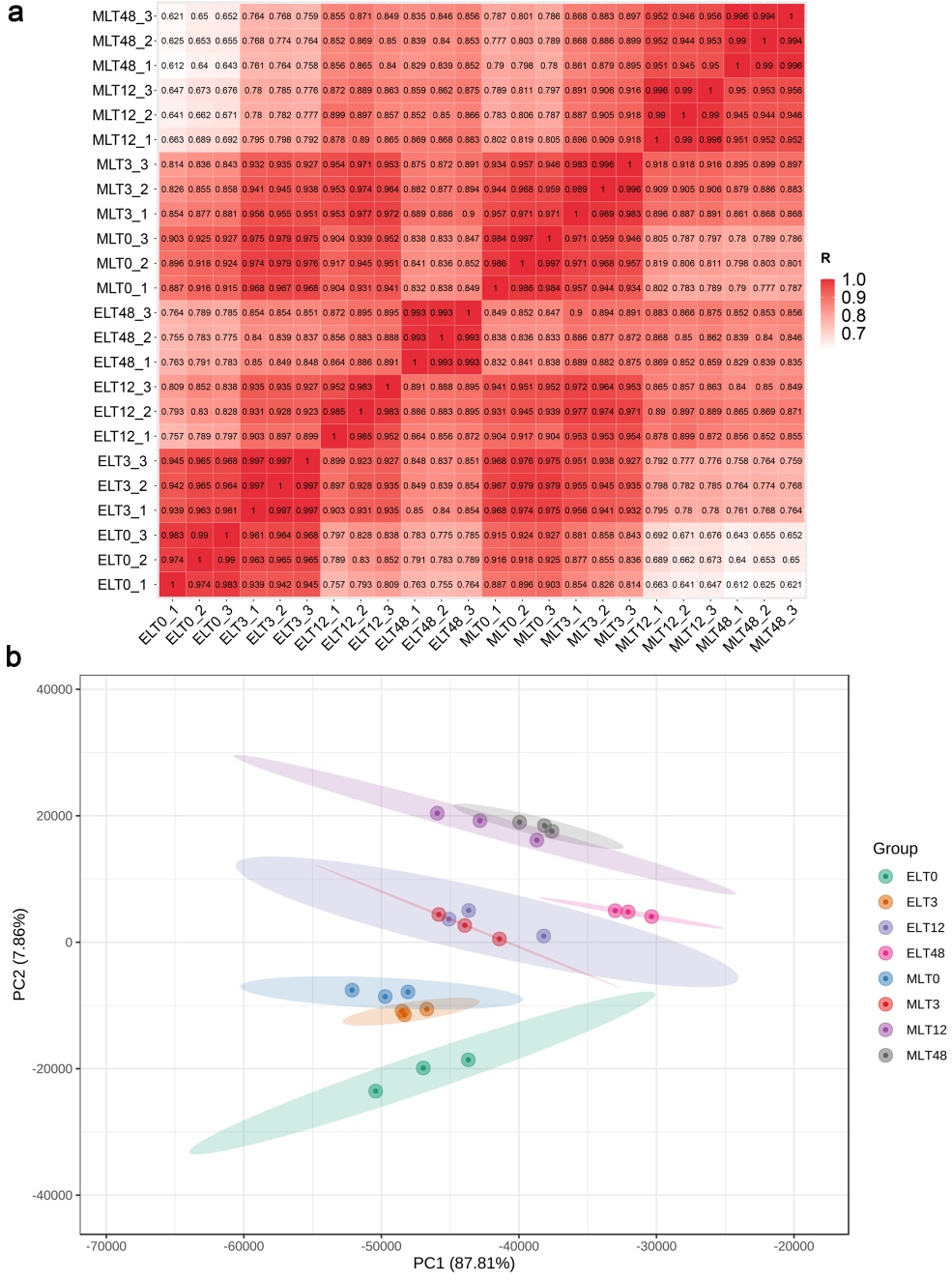


**Fig. S2** Pearson correlation (**a**) and PCA plot (**b**) of gene expression (FPKM values) among 24 RNA-seq samples after 0 h, 3 h, 12 h and 48 h of MLT and ELT

**
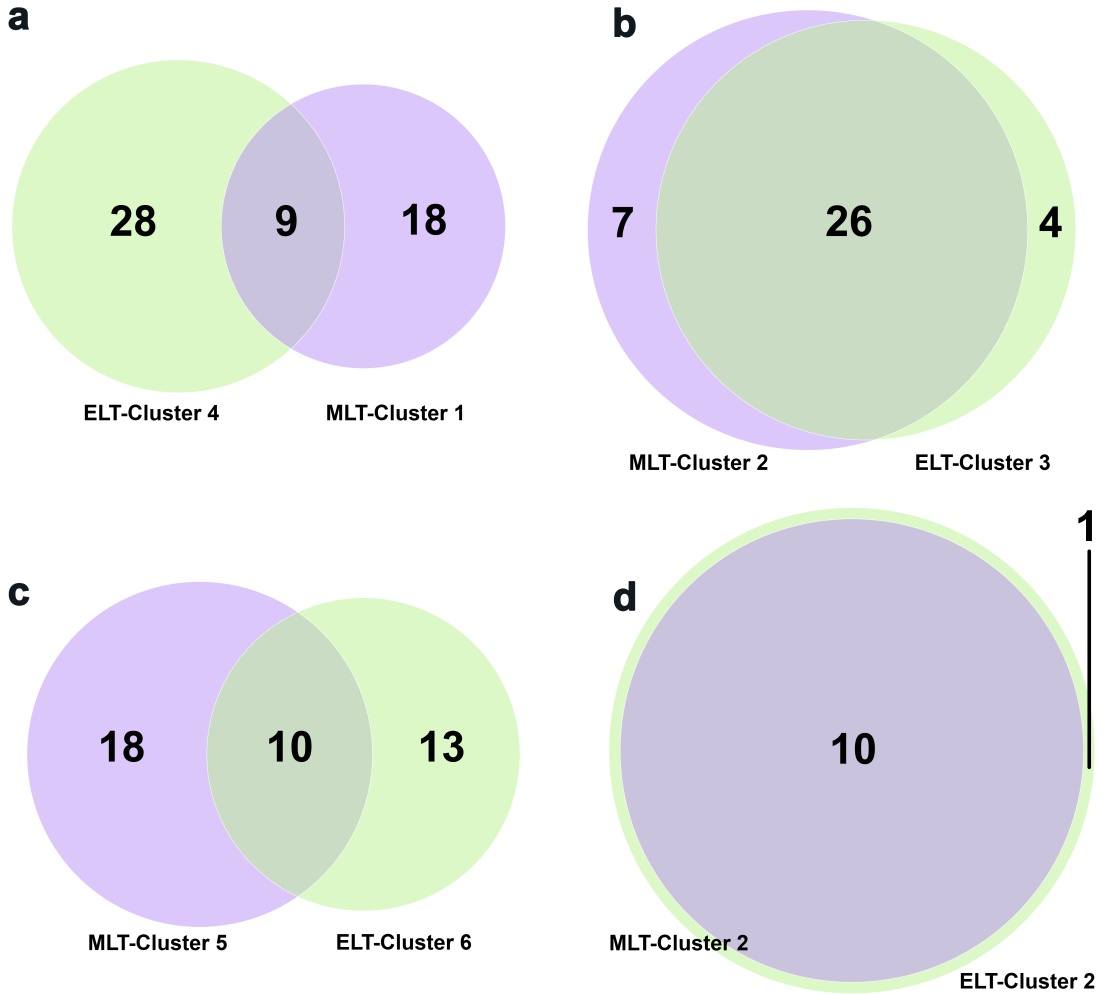
**

**Fig. S3** Venn diagram analysis. **a** The counts of intersection elements within the DEGs datasets related to ‘Glycosyltransferase’ pathway between ELT Cluster 4 and MLT Cluster 1 were shown; **b** The counts of intersection elements within the DEGs datasets related to ‘Galactose metabolism’ pathway between ELT Cluster 3 and MLT Cluster 2 were displayed; **c** The counts of intersection elements within the DEGs datasets related to ‘Starch and sucrose metabolism’ pathway between ELT Cluster 6 and MLT Cluster 5 were presented; **d** The counts of intersection elements within the DEGs datasets related to ‘tyrosine decarboxylase activity/ aromatic-L-amino-acid decarboxylase activity’ term between ELT Cluster 2 and MLT Cluster 2 were exhibited

**Table S3** Primers of candidate genes for qRT-PCR

| Gene ID | Gene annotation | Primers (5'-3') |
| --- | --- | --- |
| TRINITY_DN16804_c0_g1 | Mitogen-activated protein kinase kinase kinase 18 *MAPKKK18* | F: TTGTTTGTTTTACACCTCTGTTTCT;  R: ATCAATGGAGGTAATCCAGAACTCT |
| TRINITY_DN24048_c2_g2 | Calcium-dependent protein kinase 9 *CDPK9* | F: TGGACACAGATAATGATGGAAGAAT;  R: TTCAATCAGTGCATTAACTTTGTGT |
| TRINITY_DN19692_c0_g2 | Dehydration responsive element binding protein 2A *DREB2A* | F: GATTTTGGCTTGGAATTCTTGAGAC;  R: ATTCTCTTTTCAATTACAGCACGGC |
| TRINITY_DN22269_c1_g3 | Inducer of CBF expression 1 *ICE1* | F: CAGGTCTCTTGCTCTCCACTATGAG;  R: GGATAGCTTGCAGGAAAATGAATAT |
| TRINITY_DN12533_c2_g2 | Chlorophyll a-b binding protein CP24 10B *LHCB4B* | F: TTTTTGTTCAATTTCCAATCTTCCT;  R: TTAGCCCCGAGATAATTCAAGATTC |
| TRINITY_DN16555_c18_g1 | Light-harvesting complex II chlorophyll a/b binding protein 23 *LHCB23* | F: ACCTCAAGATCCCTGTTCTTGGC;  R: TGGGACCCTTCTCATCTCAGACT |
| TRINITY_DN20332_c1_g1 | Catalase-1/2 *CAT1/2* | F: AGAATGAACAGCTTGCATTTGGC;  R: ATCTGGGAGCTGCAGATAGTTTG |
| TRINITY_DN22437_c2_g2 | UDP-glucose 6-dehydrogenase 2 *UGDH2* | F: CAATACTCCCACCAAGACTCGT;  R: TTCTATGGCCTCAGCTGTTTTG |
| TRINITY_DN20650_c0_g1 | Trehalose-phosphate synthase 11 *TPS11* | F: CATGGCAAAATATTACCTTGACGAT;  R: AACTAGATCAAGCTTCAGTTTGTGC |
| TRINITY_DN22076_c1_g1 | Xyloglucosyl transferase 15 *XTH15* | F: CAGAAACAGGTTGAGATGGGTCC;  R: GAACAACCGCAAATTGCAAGATC |
| /  According to Su et al (2023) | Tubulin beta-5 chain *TUB5* | F: GCTGCTGTTACGGTATGTTTGTGT;  R: ATAGTGGACCCTTTGATCGACCT |

Su J, Xian KH, Fu CM, He JX, Liu BJ, Huang NZ. Selection of suitable reference genes in *Paulownia fortunei* (Seem.) Hemsl. under different tissues and abiotic stresses for qPCR normalization. Czech J Genet Plant Breed. 2023;59(4):205-18
